# Genomic landscape and distinct molecular subtypes of primary testicular lymphoma

**DOI:** 10.1186/s12967-024-05140-8

**Published:** 2024-05-01

**Authors:** Weilong Zhang, Ping Yang, Yaru Yang, Shuozi Liu, Yongdeng Xu, Chaoling Wu, Jing Wang, Cuiling Liu, Hui Liu, Shuangshuang Li, Wei Huang, Hongmei Jing

**Affiliations:** 1 Department of Hematology, Lymphoma Research Center, Peking University Third Hospital, Beijing, 100191 China; 2 Department of Pathology, School of Basic Medical Sciences, Peking University Health Science Center, Beijing, 100191 China; 3 Department of Hematology, Beijing Hospital, National Center of Gerontology, Beijing, 100005 China; 4 MyGenostics Inc, Beijing, 101300 China

**Keywords:** Primary testicular lymphoma, Molecular subtype, Genetic variants, HLA, Immune escape

## Abstract

Primary testicular lymphoma (PTL) is a rare lymphoma predominantly occurring in the elderly male population. It is characterized by a limited response to treatment and a heightened tendency towards relapse. Histologically, approximately 90% of PTL cases are classified as diffuse large B-cell lymphomas (DLBCL). Genetic features of PTL were delineated in a limited scope within several independent studies. Some of the articles which analyzed the genetic characterization of DLBCL have incorporated PTL samples, but these have been constrained by small sample sizes. In addition, there have been an absence of independent molecular typing studies of PTL. This report summarizes the common mutational features, copy number variations (CNVs) and molecular typing of PTL patients, based on whole-exome sequencing (WES) conducted on a cohort of 25 PTL patients. Among them, HLA, CDKN2A and MYD88 had a high mutation frequency. In addition, we found two core mutational characteristics in PTL including mutation in genes linked to genomic instability (TP53 and CDKN2A) and mutation in immune-related genes (HLA, MYD88, CD79B). We performed molecular typing of 25 PTL patients into C1 subtype with predominantly TP53 mutations and C2 subtype with predominantly HLA mutations. Notably, mutations in the TP53 gene predicted a poor outcome in most types of lymphomas. However, the C1 subtype, dominated by TP53 mutations, had a better prognosis compared to the C2 subtype in PTL. C2 subtype exhibited a worse prognosis, aligning with our finding that the mechanism of immune escape in PTL was primarily the deletions of HLA rather than PD-L1/PD-L2 alterations, a contrast to other DLBCLs. Moreover, we calculated the tumor mutation burden (TMB) and identified that TMB can predict prognosis and recurrence rate in PTL. Our study underscores the significance of molecular typing in PTL based on mutational characteristics, which plays a crucial role in prognostication and guiding therapeutic strategies for patients.

**Supplementary Information:**

The online version contains supplementary material available at 10.1186/s12967-024-05140-8.

## Introduction

PTL is an extremely rare and aggressive tumor, mainly occurring in older males. Histologically, about 90% of PTL cases are classified as diffuse large B-cell lymphomas (DLBCL). Immunohistochemical analyses and DNA microarray studies have indicated that 60% to 90% of PTL-DLBCL cases belong to the activated B-cell-like (ABC) subtype. Approximately 40% of PTL cases have the propensity to disseminate to extranodal organs, including central nervous system (CNS), the contralateral testis, skin, lung and other soft tissue [1–4]. The occurrence of PTL in what is considered an immune-privileged site contributes to its poor responsiveness to various treatment modalities and a relatively high rate of recurrence [2, 5, 6].

The negative prognosis and high recurrence rate of PTL are associated with immune escape mechanisms, including activation of immune checkpoints and loss of human leukocyte antigen (HLA). Programmed cell death 1 (PD-1) and cytotoxic T lymphocyte-associated Antigen-4 (CTLA-4) are currently the most investigated immune checkpoints. PD-L1, a ligand protein produced by tumor cells, can bind to PD-1 of T lymphocytes. The combination between PD-L1 and PD-1 induces deactivation, apoptosis, and depletion of T cells, which in turn inhibits the activation, proliferation and anti-tumor function of tumor antigen-specific CD8 + T cells. PD-L1/PD-L2 copy number alterations and additional translocations at relevant sites are common in PTL. These changes are thought to be responsible for immune escape in PTL [7–10]. CTLA-4 plays a crucial role in modulating T cell activation and tolerance [11, 12]. Another mechanism of tumor escape is the loss of HLA. Deficiencies in both HLA class I (HLA-A, B, C) and class II (HLA-DR, DQ, DP) can lead to inherently weak anti-tumor immune responses and undermine the efficacy of T cell-targeted immunotherapies. In addition, loss of HLA I and II expression significantly contributes to the phenomenon of immune privilege [13, 14]. In a study by Chapuy et al. rearrangements of PD-L1/PD-L2 in PTL were found to be prevalent in over 50% of PTL cases [15]. Contrastingly, Minderman's study revealed expression of PD-L1 or 9p24.1/PD-L1/2 CNA was detected in only a small number of samples. In addition, their team extracted PD-L1 messenger RNA (mRNA) expression data from publicly available databases for validation, and they found that there was no enhancement of PD-L1 expression in PTL compared to primary mediastinal B-cell lymphomas (PMBCLs) [16–19]. Instead, HLA deletions were common in their PTL cohort. Their article suggests that the mechanism of immune escape in PTL and CNS lymphoma is primarily HLA loss rather than the use of the PD1/PD-L1/2 checkpoint [20].

A limited number of prior studies focusing exclusively on PTL, as well as investigations of DLBCL that include PTL cases, have identified a range of genetic alterations in PTL patients [1, 15, 21–25]. High-frequency of somatic mutations in the MYD88 and CD79B genes (NF-κ-B pathway-associated genes) have been shown to be broadly present in PTL. Additionally, previous studies have also demonstrated the presence of BCL2 and BCL6 translocations in PTL [22, 26, 27].

Currently, both independent studies on PTL and those incorporating PTL within broader DLBCL research have been limited by small sample sizes. And there has been a lack of systematic molecular typing studies in PTL. Here, we performed whole exome sequencing (WES) analysis on 25 PTL patients, enabling us to perform mutation typing in this group. We anticipate that our molecular typing approach will shed light on the molecular heterogeneity of PTL, thereby facilitating more precise, subtype-specific treatments. Moreover, we calculated the tumor mutation burden (TMB) and established its potential as a prognostic and recurrence predictor in PTL.

## Methods

### Patients and samples

Formalin-fixed, paraffin-embedded specimens of 25 PTL patients (14 cancer samples and matched normal tissues, 11 cancer samples) were obtained and identified at the time of diagnosis. We statistically analyzed the clinical information of 25 PTLs, including age, histologic type, pathology, stage, B-symptoms, ECOG, IPI and recurrence (Table 1). In each case, the diagnosis of PTL was established employing appropriate diagnostic criteria (WHO classification) of lymphoid tumors with combinations of histologic, immunohistochemical, flow cytometric, and genetic evaluation (Additional file 12: Table S1). The study protocols described above comply with the ethical requirements of Peking University Third Hospital.

**Table 1:** The clinical information of 25 PTL patients

	Total
Age	
Mean (SD)	63.7 (± 12.1)
Diagnose	
DLBCL	25 (100%)
Pathology	
nonGCB	22 (88.0%)
GCB	3 (12.0%)
Stage	
I	3 (12.0%)
II	7 (28.0%)
III	2 (8.0%)
IV	13 (52.0%)
B symptom	
Yes	5 (20.0%)
No	20 (80.0%)
ECOG	
0	20 (80.0%)
1	3 (12.0%)
2	1 (4.0%)
4	1 (4.0%)
IPI	
0	2 (8.0%)
1	1 (4.0%)
2	12 (48.0%)
3	6 (24.0%)
4	3 (12.0%)
5	1 (4.0%)
Recurrence	
Yes	9 (36.0%)
No	16 (64.0%)

### Whole exome sequencing and quality control of raw sequencing data

We used the traditional CTAB method to extract genomic DNA from tumor samples, followed by quality assessment of the extracted DNA using agarose gel electrophoresis and Nanodrop. The quality-checked genomic DNA was randomly broken into 180–280 bp fragments using a Covaris fragmentation instrument. Subsequently, DNA libraries were prepared and whole exome capture was conducted via hybridization with biotinylated single-strand DNA capture probe. Post-hybridization, the libraries underwent PCR amplification and quality control. Libraries passing quality checks were sequenced on the Novaseq platform. The read length of this this sequencing is 2*150 bp. The highest read depth is 155.24, the lowest read depth is 32.91, the average read depth is 72.98.

In the high-throughput sequencing data, a minor fraction of reads may contain low-quality bases or undetected bases (N). To ensure the quality of information analysis, raw reads were subjected to quality controlled using the Fastp, resulting in clean reads [28]. These clean reads were aligned to the reference genome (version hg19) using the software bwa to generate alignment data [29]. And subsequent analyses were performed based on these clean reads. The quality control process involved trimming low-quality bases from both the 5' and 3' ends of the sequences, discarding sequences with a high proportion of undetected bases (N), and removing adaptor sequences from the reads.

### Somatic SNVs/Indels variant detection

Somatic mutation detection was performed using the Mutect2 module in GATK to obtain information on SNVs/Indels variants [30]. The results of Mutect2 were annotated through the simultaneous association with multiple databases (e.g. dbSNP, 1000g, ESP6500, HGMD, OMIM, etc.) using the software ANNOVAR [31]. To ensure high-quality somatic mutation data, further filtration was applied based on specific criteria: (a) Mut_ratio > 5%, Alt >  = 3 and Depth >  = 20; (b) remove the results with SNP effect of unknown and nonframeshift and keep the results with gene region of exonic and splicing; (c) keep the results with mutation frequency below 0.001 in 1000g, ESP6500 and ExAC databases; (d) remove mutation results annotated to interfering genes. Heat maps representing SNV and CNV mutation results were generated using the ComplexHeatmap R package [32]. Additional analyses, including the statistics of SNV mutation patterns, TMB statistics calculation, survival analysis, forest plotting, sample clustering analysis, and CNV mutation frequency statistics on samples were performed using the R language internal script (version 4.1.0).

### CNV variant detection

For CNV detection in our samples, we employed CNVkit software, which determines copy number changes by comparing the depth distribution of sequencing reads to that of a reference genome [33]. The occurrence of CNVs in specific genes was subsequently analyzed for each sample using the CNVRanger package in R [34].

### Pathway enrichment analysis

Pathway enrichment analysis was performed using Reactome to elucidate the functions of genes undergoing SNV mutations [35]. And the relationship network between pathways and genes was visualized using Cytoscape [36].

### Gene expression data and analysis

To further our research, we accessed gene expression arrays for 22 cases of PTL and 232 nodal DLBCLs from the NCBI Gene Expression Omnibus (GEO) database (GSE10524, GSE10846, GSE61578) [1, 15, 37]. Gene expression levels were quantified from these microarray data using the robust multiarray averaging (RMA) algorithm. These expression levels were then transformed using a log2 scale for enhanced analytical clarity.

## Result

### Genetic characteristics and signaling pathways of 25 PTL patients

**Fig. 1:**
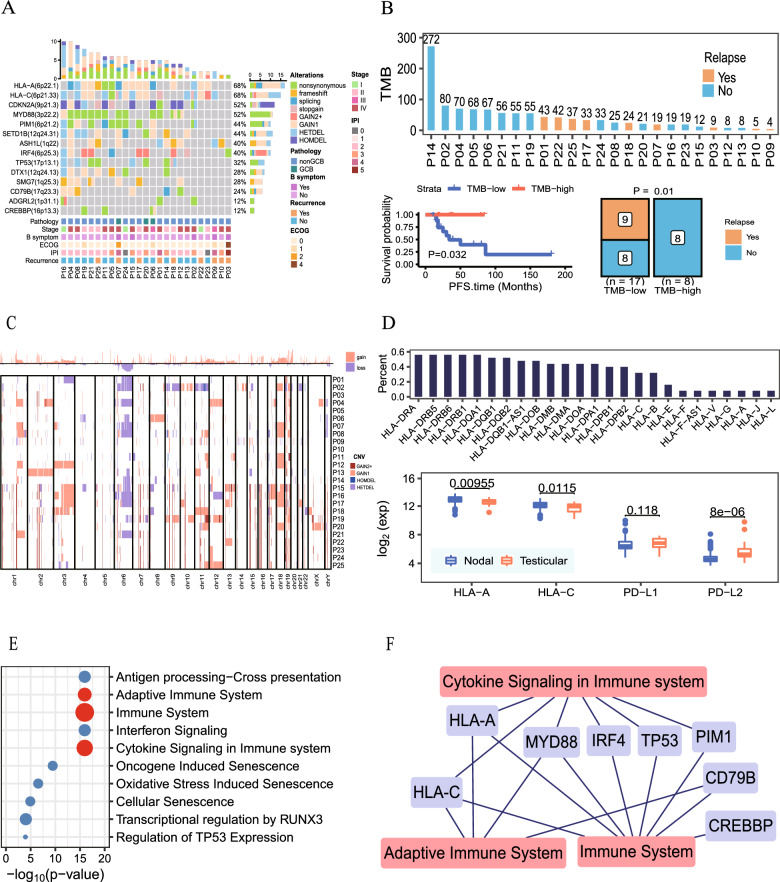
Genetic characteristics and signaling pathways of 25 PTL patients through whole-exome sequencing. **A** The SNV and CNV spectrum of 25 PTL patients, showing the mutation frequency of each gene (right) and the clinical data of each sample (bottom). **B** Relationship between relapse and TMB in 25 PTL patients. The Kaplan–Meier curves for PFS of 25 PTL patients in TMB (log-rank test, P = 0.032) and the relapse rates in the TMB-high and TMB-low groups (Wilcoxon test, P = 0.01). **C** CNV profiles on all chromosomes of 25 PTL patients. **D** Proportion of CNV deletions in each type of HLA. The expression of HLA-A, HLA-C, PD-L1 and PD-L2 between nodal DLBCLs and PTL (Wilcoxon test, HLA-A, P = 0.00955; HLA-C, P = 0.0115; PD-L1, P = 0.118; PD-L2, P = 8e−06). **E** Pathway analysis of mutational genes in the exome of 25 PTL patients. **F** A network of genes enriched in three immune-related pathways

Based on the SNV and CNV profiles of 25 PTL patients, we can find that both HLA-A and HLA-C exhibit an overall mutation rate of 68% and the total mutation rate of HLA is as high as 84% (Fig. 1A). Although previous studies have reported CNV deletion in HLA-C. However, it is noteworthy that we also identified somatic mutations in HLA-C in PTL, which had not been reported in previous studies. 4 out of 25 patients had mutations in HLA-C, three of which were stop gain alterations. All three stop-gains are on the second exon of the transcript. The change of its cDNA from C to T at position 232 caused a change in the amino acid of the protein sequence at position 78 from glutamine (Q) to stop-gains. One of the nonsense mutations was in exon 4 of the transcript. Its cDNA was changed from A to G at position 749, causing a change in amino acid from glutamine (Q) to arginine (R) at position 250 of the protein sequence (Fig. 1 and Additional file 11: Table S4). These mutations lead to changes in the encoded proteins and thus loss of function. Moreover, we discovered that somatic mutations in HLA-C, as well as somatic mutations in ASH1L, contributed to worse prognosis for PTL patients (Additional files 2, 3: Figs. S2, S3). We also found that the CDKN2A gene had the third-highest mutation rate at 52% in 25 PTL patients (Fig. 1A and Additional file 1 Fig. S1A). CNV deletions in CDKN2A primarily manifested as homozygous deletions in seven out of the 25 PTL patients, corroborating previous findings by Chapuy et al. [15]. Analysis of the genes on chromosome 9 reveals a very large proportion of deletions in CDKN2A and CDKN2B (Additional file 7: Fig. S7A). Compared with nodal DLBCLs, the expression of CDKN2A on PTL patients is significantly low (Additional file 11: Fig. S11A, Wilcoxon test, P = 5.8e–06). In addition to the deletion of CDKN2A in PTL patients, which has been reported to be associated with genomic instability, we also identified deletions and mutations in TP53, another gene associated with genomic instability in PTL patients. We found that 4 patients with PTL had abnormal CNV profiles in TP53, with three presenting heterozygous deletions and one demonstrating both heterozygous deletions and mutations (Fig. 1A).

Then we performed TMB in 25 PTL patients and subsequently found out that high TMB predicted better progression-free survival (PFS) and overall survival (OS) time (Fig. 1B and Additional file 4: Fig. S4A, PFS, P = 0.032; OS, P = 0.041). Additionally, patients with high TMB had a low relapse rate (Fig. 1B and Additional files 5, 6: Figs. S5, S6, Chi-square test, P = 0.01).

### CNVs profiles in PTL

As for CNV profile, we can find a substantial number of deletions on chromosome 6 where HLA locates (Fig. 1C). In addition to the high-frequency deletions of HLA class I genes, including HLA-A, HLA-B, HLA-C, HLA-E, HLA-F and HLA-G, we also found high-frequency deletions of CNV in HLA class II genes (Fig. 1D and Additional file 7: Fig. S7A). Notably, the expression of HLA-A and HLA-C in PTL is lower than that in nodal DLBCLs. However, compared with the expression of PD-L1 in nodal DLBCLs, the expression of PD-L1 did not decrease in PTL patients. And the expression of PD-L2 in PTL patients was higher than that in nodal DLBCLs (Fig. 1D, Wilcoxon test). Compared with HLA genes expression on nodal DLBCLs, the expression of HLA genes was decreased in PTL patients (Additional file 8: Fig. S8A). Furthermore, we identified genes displaying deletions such as P4HTM and WDR6, which were associated with poor prognosis for PTL patients (Fig. 1C and Additional file 9 Fig. S9, log-rank test). Additionally, amplified genes in PTL patients such as TLE1 and ADAMTSL4-AS1 also contributed to the unfavorable prognosis of PTL patients (Additional file 10: Fig. S10, log-rank test). Besides, pathway analysis of the mutant genes in these 25 PTL samples revealed significant enrichment in pathways related to the immune system, antigen processing cross-presentation, and signal transduction (Figs. 1E–F).

### Molecular subtyping of 25 PTL patients

**Fig. 2:**
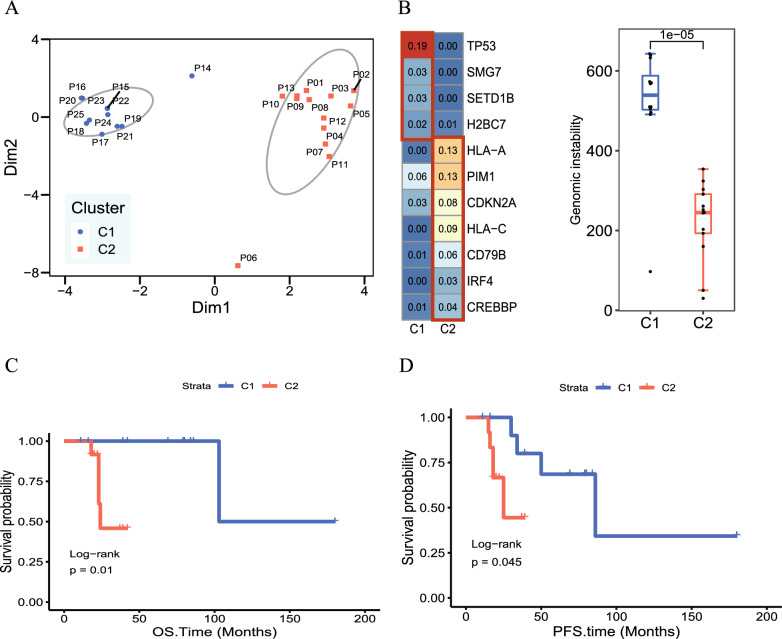
Molecular subtyping of 25 PTL patients. **A** 25 PTL patients are divided into two subtypes. **B** The total counts of genes with CNV in C1 and C2 (Student’s t test, P = 0.00021). **C** The Kaplan–Meier curves for OS (log-rank test, P = 0.01) of C1 and C2 subtypes. **D** The Kaplan–Meier curves for PFS (log-rank test, P = 0.045) of C1 and C2 subtypes

We performed a cluster analysis of the 25 PTL patients based on the results of SNV, CNV and TMB profiles, which divided the patients into two distinct subtypes (Fig. 2A). C1 is a genomic-instability subtype characterized primarily by the mutation of TP53. C2 is an immune-escape subtype characterized by the mutation of immune-related genes such as HLA-A, HLA-C, PIM1 and so on, with HLA genes having the highest frequency. Moreover, the total counts of gene with CNV in C1 is higher than that in C2, signifying a significantly greater degree of genomic instability in C1 (Fig. 2B). Interestingly, despite TP53 mutations typically predicting poorer prognosis in lymphomas, C1, predominantly featuring TP53 mutations in PTL, exhibits improved PFS and OS (Fig. 2C, OS, P = 0.01, PFS, P = 0.045). By comparing the clinical information between C1 and C2, we found that there was only a significant difference in stage between the two groups. Patients in C2 were basically at stage III-IV when they were diagnosed (Table 2).

**Table 2:** Different clinical features of PTL patients in C1 and C2 subtypes

	C1	C2	P-value
Age			
Mean (SD)	63.9 (± 9.8)	63.5 (± 14.4)	0.94
Diagnose			
DLBCL	12 (100.0%)	13 (100.0%)	NA
Pathology			
nonGCB	11 (91.7%)	11 (84.6%)	1.00
GCB	1 (8.3%)	2 (15.4%)	
Stage			
I	3 (25.0%)	0 (0.0%)	< 0.001
II	7 (58.3%)	0 (0.0%)	
III	0 (0.0%)	2 (15.4%)	
IV	2 (16.7%)	11 (84.6%)	
B symptom			
Yes	1 (8.3%)	4 (30.8%)	0.37
No	11 (91.7%)	9 (69.2%)	
ECOG			
0	11 (91.7%)	9 (69.2%)	0.48
1	1 (8.3%)	2 (15.4%)	
2	0 (0.0%)	1 (7.7%)	
4	0 (0.0%)	1 (7.7%)	
IPI			
0	2 (16.7%)	0 (0.0%)	0.22
1	1 (8.3%)	0 (0.0%)	
2	6 (50.0%)	6 (46.2%)	
3	3 (25.0%)	3 (23.1%)	
4	0 (0.0%)	3 (23.1%)	
5	0 (0.0%)	1 (7.7%)	
Recurrence			
Yes	4 (33.3%)	5 (38.5%)	1.00
No	8 (66.7%)	8 (61.5%)	

## Discussion

Several studies in recent years have delved into the mechanisms underlying immune escape in PTL and to elucidate the factors contributing to its poor response to treatment and prognosis. In addition, there have been a few studies on the mutation spectrum of PTL genes, but there are currently no studies that characterize the mutation profiles of PTL patients in order to perform typing and prognostic analyses. Such a clinically applicable typing approach we have created could stratify PTL, thereby guiding the precise treatment of patients. Here, we performed the WES on 25 PTL patients to delineate the mutation characteristics, chromosomal rearrangement, and CNVs in PTL, ultimately classifying these patients into two molecular subtypes.

By analyzing the mutation spectrum of PTL, we can find that HLA-A, HLA-C, CDKN2A, MYD88, PIM1, SETD1B, ASH1L, IRF4, TP53, DTX1, SMG7, CD79B, ADGRL2 and CREBBP exhibit high mutation rates in PTL. Among them, HLA-A, HLA-C, PIM1 and MYD88 are immune-related genes. In addition to mutations in immune-related genes, we identified a high frequency of mutations in genes affecting genomic stability, such as CDKN2A, TP53, SETD1B and SMG7. Genomic instability is often caused by DNA mutations and aberrant epigenetic modifications (DNA methylation and histone modifications) [38–44]. Many studies have demonstrated that genetic alterations in TP53 and CDKN2A can induce or tolerate genomic instability [45–52]. In early deep sequencing analyses, somatic mutations in TP53 were seen in only 20% of DLBCL. Somatic mutations in TP53 were even rarer in PTL [15, 21]. However, in our cohort, we found TP53 somatic mutations in 24% of PTLs, including four nonsynonymous as well as one stop-gain. In our subsequent molecular typing of PTL, somatic mutations in TP53 were a major feature present in one of the subtypes. We also found a high frequency of homozygous deletions in the CDKN2A gene. This was a more characteristic genetic manifestation of PTL compared to DLBCL [15].

Currently there is no consensus in the study of PTL regarding its main immune escape mechanism. Some scholars consider the gain of PD-L1/PD-L2 in tumors as the main immune escape mechanism of PTL. Alterations in PD-L1/PD-L2 (9p24.1) copy number were common (> 50%) in PTL in their studies [15, 53, 54]. Other scholars have suggested that HLA deletion is the main immune escape mechanism in PTL. They found a high frequency of HLA I and II deletions in PTL but little expression of PD-L1, resulting in no 9p24.1/PD-L1/2 CNA [1, 55]. Our analysis of PTL by whole exome sequencing suggests that the main tumor immune escape mechanism in PTL is HLA deletion on rather than PD-L1/PD-L2 alteration. We found a large number of deletions at the chromosome 6 locus, with very frequent expression deletions in HLA I and HLA II. Compared to CNV in HLA I, a higher percentage of CNV deletions are found in the HLA-DR genes (HLA-DRA, HLA-DRB5, HLA-DRB6, and HLA-DRB1, etc.) and the HLA-DQ genes (HLA-DQA1, HLA-DQB1, and HLA-DQHLA-DQB2, etc.). CNV deletion of the HLA-DR genes can severely disrupts the immune response to PTL. Then we compared the CNVs of PTL patients with those of nodal DLBCLs patients for validation. Our analysis revealed that the CNVs of HLA-A and HLA-C were significantly lower in PTL patients compared with those of nodal DLBCL patients. Conversely, the CNVs of PD-L2 were significantly higher in PTL patients compared to nodal DLBCL patients. However, there was no significant difference in CNVs of PD-L1 between the two groups. These findings suggest that copy deletion at HLA loci is the main mechanism of immune escape in PTL tumors. In addition, we found somatic mutations in the HLA genes, including HLA-A and HLA-C. Among them, the mutation in HLA-C was a somatic mutation that had not been reported in previous articles. Additionally, by calculating the TMB of the PTL patients in our cohort, we found that PTL patients with higher TMB were less likely to relapse and had longer PFS. PTL patients with high TMB may indirectly reflect the tumor's ability to produce neoantigens, especially considering that most of the genes with high mutation rates in our cohort are immune-related genes. Consequently, such patients may benefit from immunotherapy, making TMB a potential indicator of treatment efficacy.

We successfully categorized PTL patients into C1 and C2 based on their mutation characteristics by cluster analysis. C1 is the subtype characterized by TP53 mutations, while C2 is the subtype characterized by mutations in immune-related genes such as HLA-A, HLA-C and PIM1. A comparison of the two subtypes revealed that C2 had worse PFS and OS compared to C1. It's worth noting that, to date, there have been no studies that have typed patients with PTL. Our mutation typing of PTL patients not only facilitates the prediction of prognosis in patients with primary treatment of PTL but also lays the groundwork for future precision medicine in PTL.

In conclusion, we found two types of core genetic variants in PTL patients including mutations in immune-related genes (HLA, MYD88, CD79B and PIM1) and genomic instability-related genes (TP53 and CDKN2A). Among them, somatic mutations in HLA-C and TP53 have hardly been identified in previous genetic characterization studies of PTL. This group has a better prognosis compared to C2. In addition, we discovered that TMB can predict prognosis and recurrence rate in PTL. Finally, PTL patients can be divided into two groups based on molecular subtyping. The first group, C1, is characterized by mutations in genes that maintain genomic stability, such as TP53. The second group, C2, is characterized by mutations in immune-related genes, including those in the HLA complex. Notably, patients within the C2 subtype exhibit a poorer prognosis, indicating a significant correlation between the prognosis of PTL patients and their immune functionality. This molecular stratification presented in our study holds potential for enhancing the precision in diagnosis, prognostic assessment, tumor staging, treatment guidance, monitoring of recurrence, and the development of targeted therapies for PTL.

### Supplementary Information

**Additional file 1: Figure S1.** Mutation spectrum of 25 PTL patients, showing the mutation frequency of each gene (right) and clinical data of each sample (bottom).

**Additional file 2: Figure S2.** Effect of mutations in HLA-C on prognosis in PTL patients. **A** The Kaplan-Meier curves for OS of HLA-C mutation (log-rank test, P=2.9e−03)

**Additional file 3: Figure S3.** Effect of mutations in ASH1L on prognosis in PTL patients. **A** The Kaplan-Meier curves for PFS of ASH1L mutation (log-rank test, P=3e−02)

**Additional file 4: Figure S4.** The Kaplan-Meier curves for OS of 25 PTL patients in TMB-low and TMB-high groups (log-rank test, P=0.041)

**Additional file 5: Figure S5.** Mutational signature analysis of 25 PTL patients. **A**–**B** Proportion of six mutation classes in the exome of 25 PTL patients. **C** Distribution of the six mutation classes in 25 PTL patients

**Additional file 6: Figure S6.** Distribution of five mutation types in 25 PTL patients

**Additional file 7: Figure S7.** The percentage of CNV and SNV profiles on chromosome. **A** The percentage of CNV and SNV profiles for multiple genes on chromosome 6. **B** The percentage of CNV and SNV profiles for multiple genes on chromosome 9

**Additional file 8: Figure S8.** Different expression of each HLA types between PTL patients and nodal DLBCLs (22 PTL patients and 232 nodal DLBCLs patients from GSE10524，GSE10846，GSE61578)

**Additional file 9: Figure S9.** Effect of deletion-based genes on prognosis in PTL patients. **A** Forest plots of deleted genes. The Kaplan-Meier curves for PFS of the CNV in P4HTM (log-rank test, P=7.6e−03) and WDR6 (log-rank test, P=7.6e−03).

**Additional file 10: Figure S10.** Effect of amplification-based genes on prognosis in patients with PTL. **A** Forest plots of amplified genes. **B** The Kaplan-Meier curves for OS of the CNV in TLE1 (log-rank test, P=9e−04) and ADAMTSL4-AS1 (log-rank test, P=3e−03)

**Additional file 11: Figure S11.** Different gene expression between PTL patients and nodal DLBCLs. (A) The expression of CDKN2A between PTL patients and nodal DLBCLs.

**Additional file 12: Table S1.** Clinical information of PTL patients in this study. **Table S2.** Sequencing fastq files quality before and after trim. **Table S3.** Map statistics for aligning to the genome. **Table S4.** SNV results after filter. **Table S5.** CNV results after CNVRanger processing. **Table S6.** Reactome enrichment results using the gene with SNV and CNV.

## Data Availability

The authors thank GSE10524, GSE10846, GSE61578 dataset from GEO database which are all available online. And the authors confirm that the data supporting the fundings of this study are available within our Additional file table.
