# Supplementary figures and images for: Genomic landscape and distinct molecular subtypes of primary testicular lymphoma
Source: J Transl Med. 2024 May 1;22:414. doi: 10.1186/s12967-024-05140-8 (PMC11064289; doi:10.1186/s12967-024-05140-8)

# Supplementary Figure 1

A

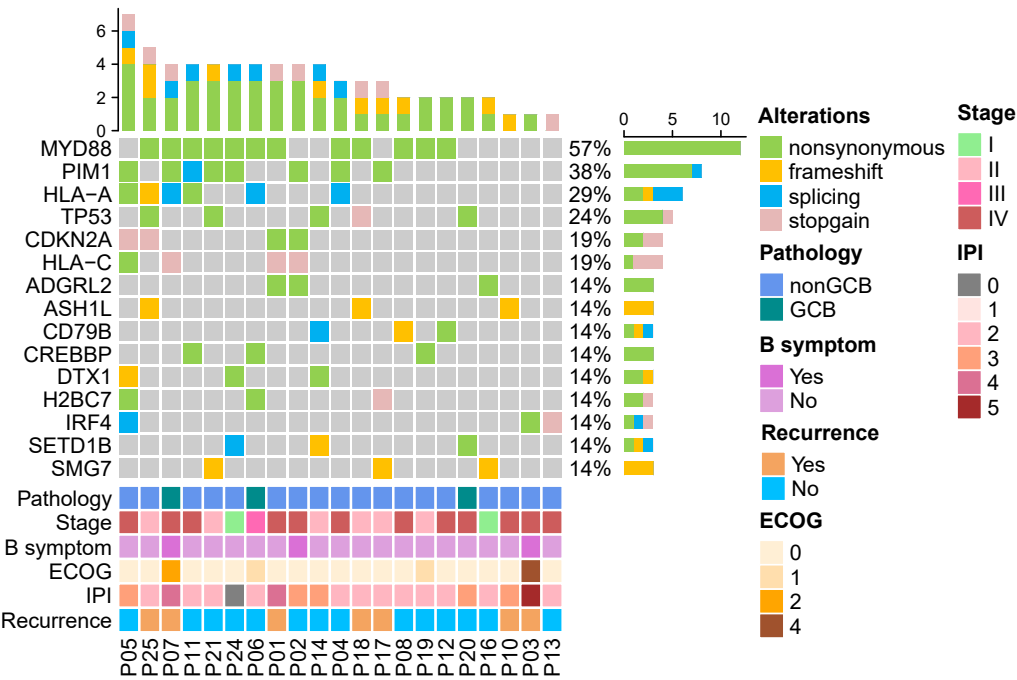

Supplement: Supplementary file 1 — Additional file 1: Figure S1. Mutation spectrum of 25 PTL patients, showing the mutation frequency of each gene (right) and clinical data of each sample (bottom). [file 12967_2024_5140_MOESM1_ESM.pdf]

## Supplementary Figure 2

A

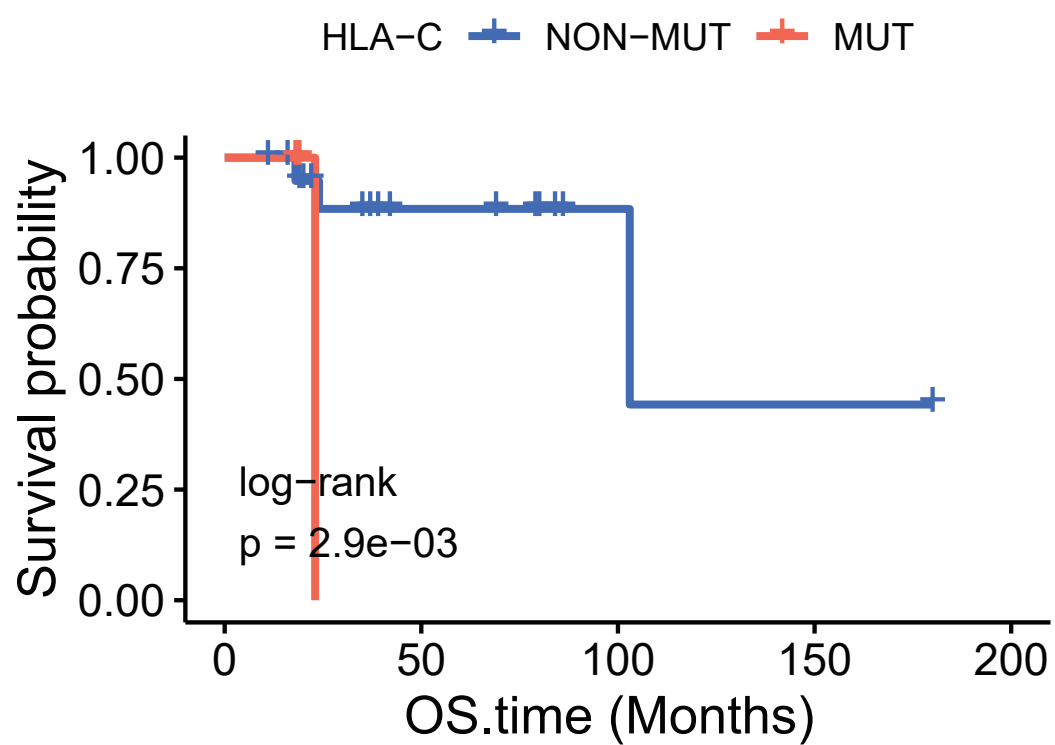

Supplement: Supplementary file 2 — Additional file 2: Figure S2. Effect of mutations in HLA-C on prognosis in PTL patients. A The Kaplan-Meier curves for OS of HLA-C mutation (log-rank test, P=2.9e−03) [file 12967_2024_5140_MOESM2_ESM.pdf]

Supplementary Figure 3

A

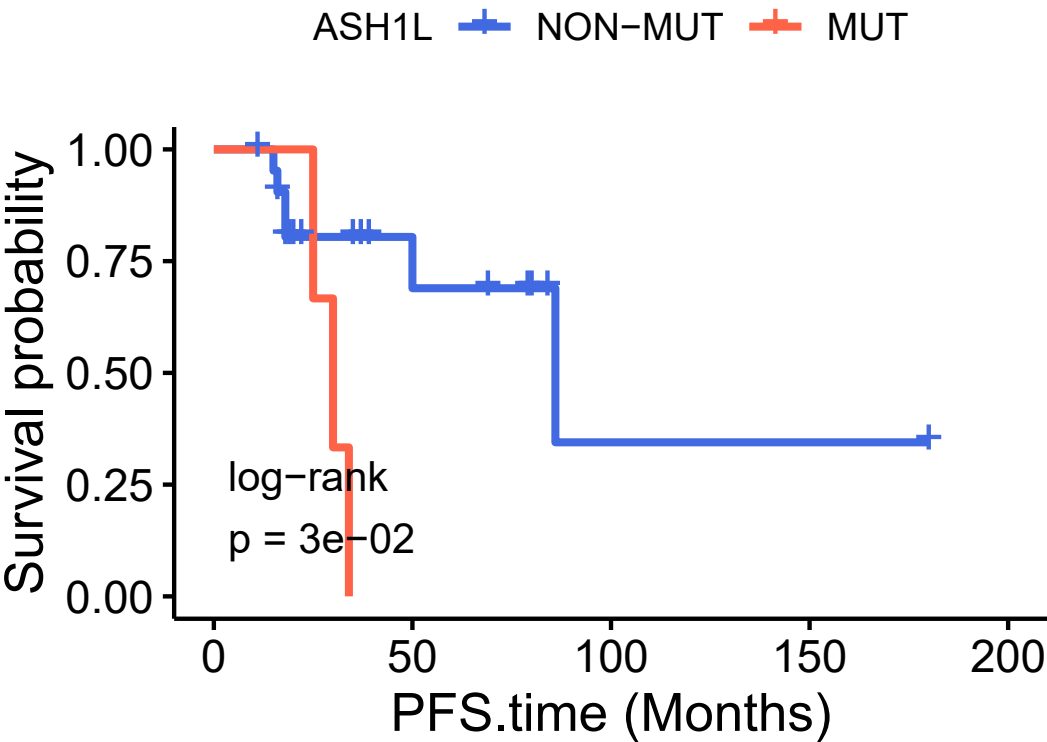

Supplement: Supplementary file 3 — Additional file 3: Figure S3. Effect of mutations in ASH1L on prognosis in PTL patients. A The Kaplan-Meier curves for PFS of ASH1L mutation (log-rank test, P=3e−02) [file 12967_2024_5140_MOESM3_ESM.pdf]

# Supplementary Figure 4

A

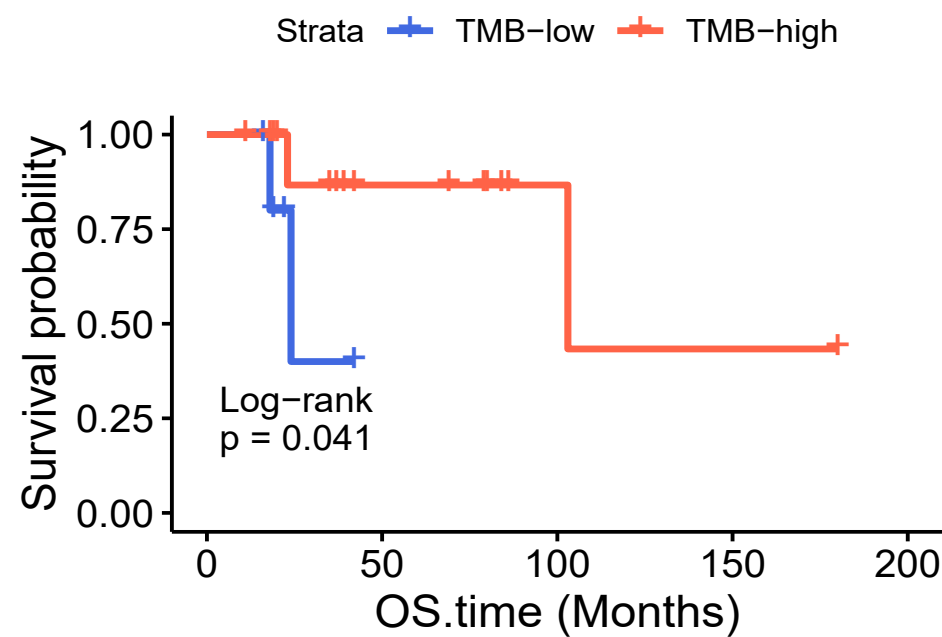

Supplement: Supplementary file 4 — Additional file 4: Figure S4. The Kaplan-Meier curves for OS of 25 PTL patients in TMB-low and TMB-high groups (log-rank test, P=0.041) [file 12967_2024_5140_MOESM4_ESM.pdf]

Supplementary Figure 5

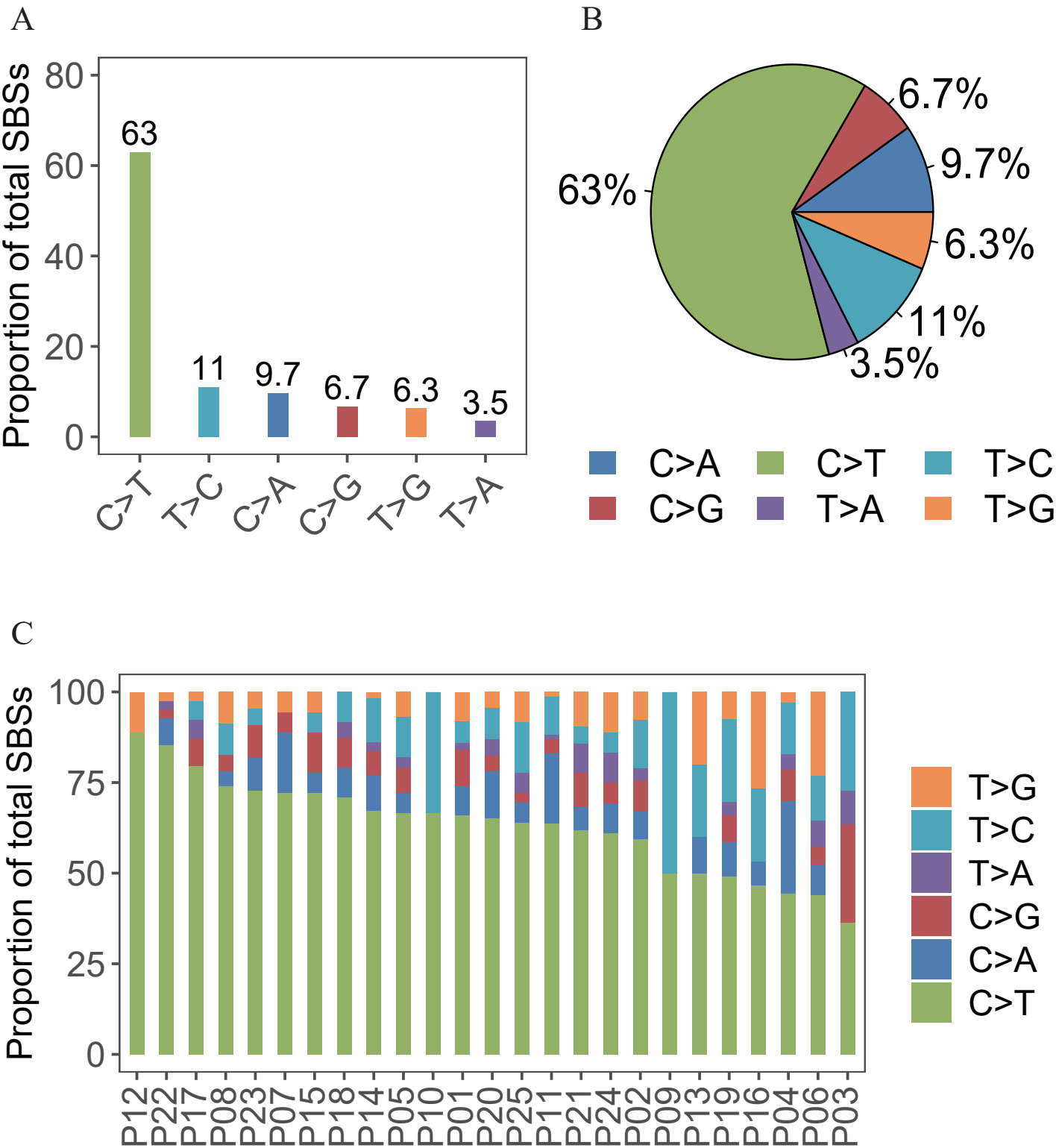

Supplement: Supplementary file 5 — Additional file 5: Figure S5. Mutational signature analysis of 25 PTL patients. A–B Proportion of six mutation classes in the exome of 25 PTL patients. C Distribution of the six mutation classes in 25 PTL patients [file 12967_2024_5140_MOESM5_ESM.pdf]

# Supplementary Figure 6

A

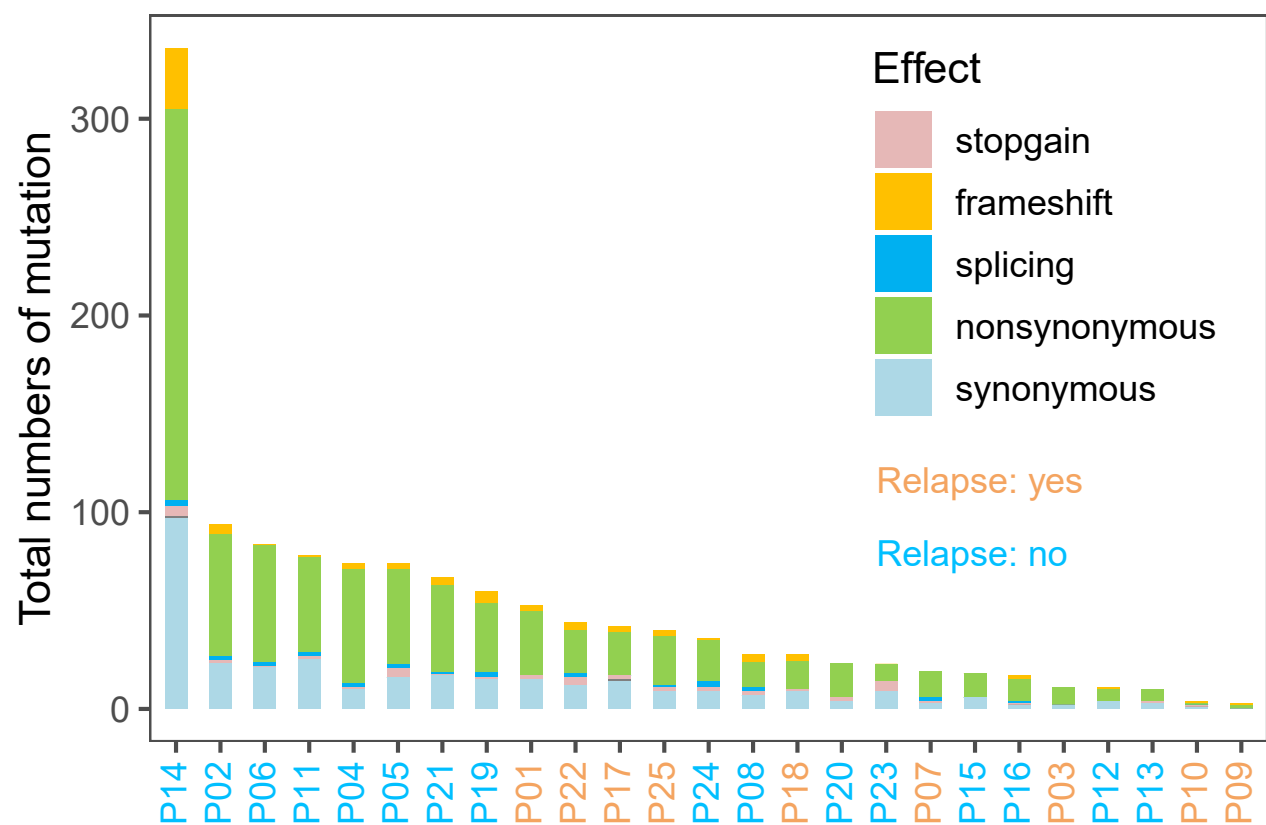

Supplement: Supplementary file 6 — Additional file 6: Figure S6. Distribution of five mutation types in 25 PTL patients [file 12967_2024_5140_MOESM6_ESM.pdf]

## Supplementary Figure 7

A

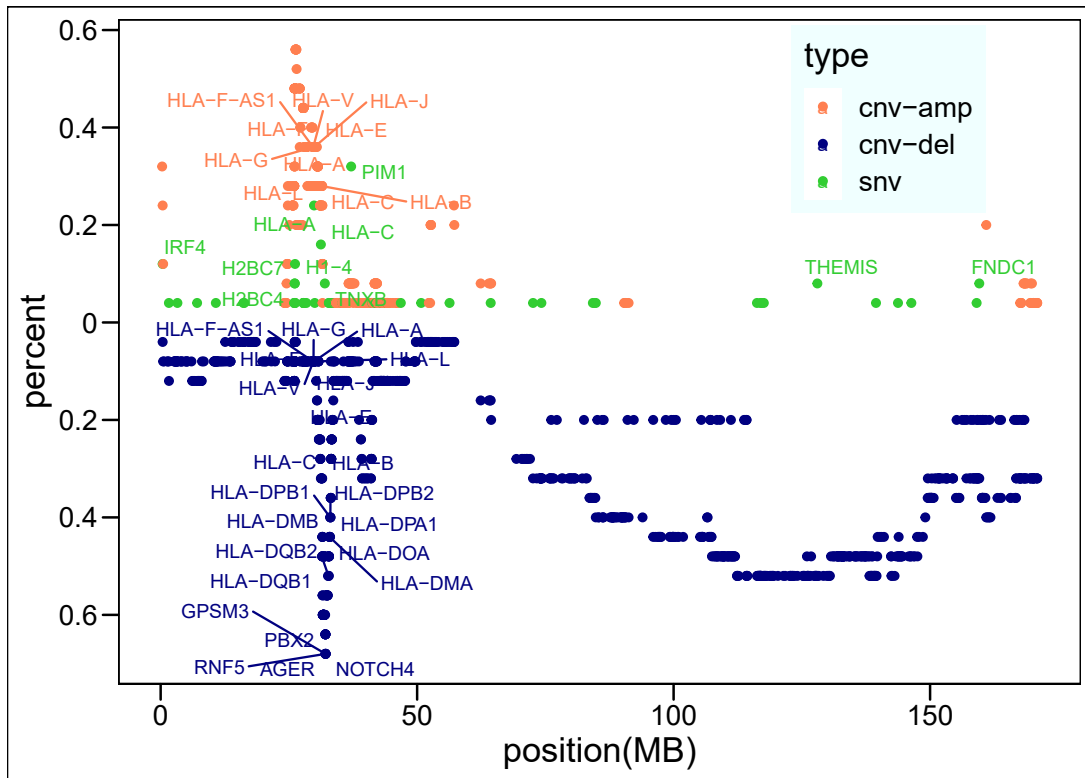

B

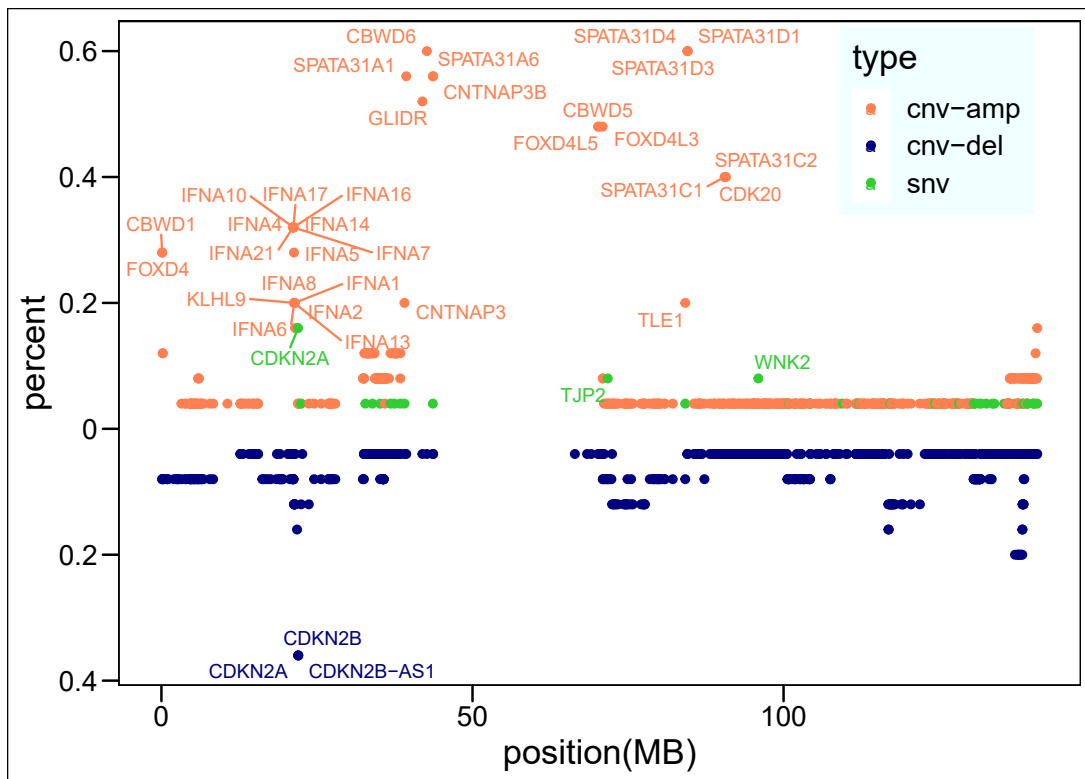

Supplement: Supplementary file 7 — Additional file 7: Figure S7. The percentage of CNV and SNV profiles on chromosome. A The percentage of CNV and SNV profiles for multiple genes on chromosome 6. B The percentage of CNV and SNV profiles for multiple genes on chromosome 9 [file 12967_2024_5140_MOESM7_ESM.pdf]

Supplementary Figure 8

A

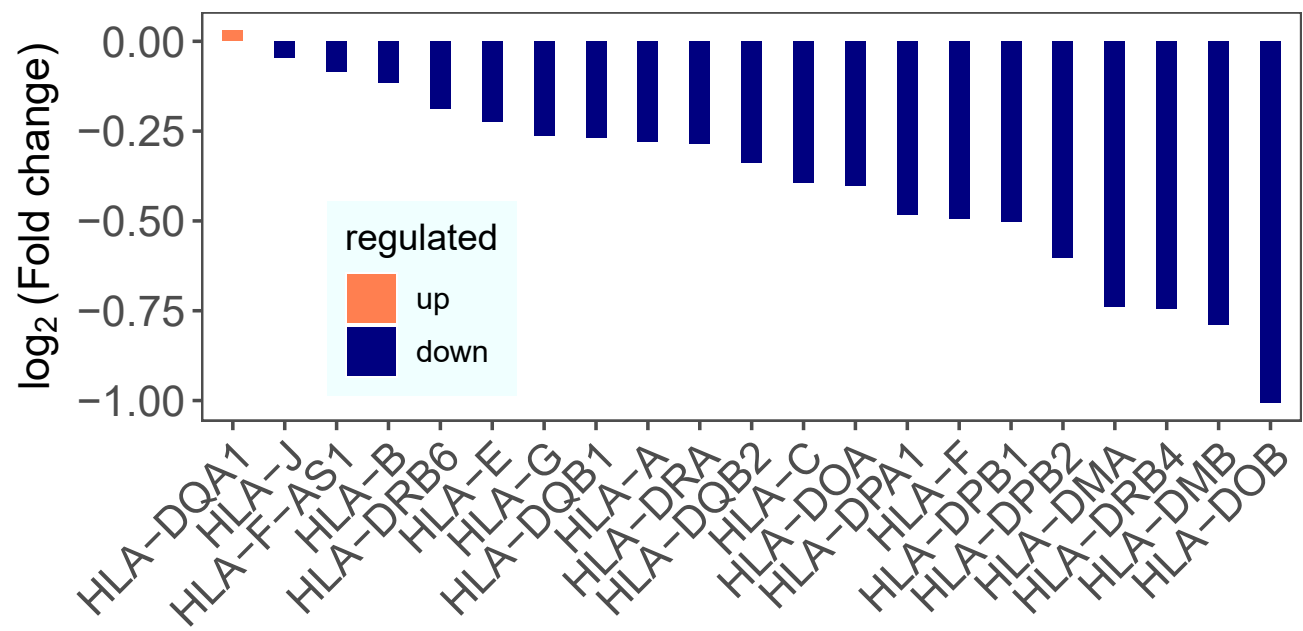

Supplement: Supplementary file 8 — Additional file 8: Figure S8. Different expression of each HLA types between PTL patients and nodal DLBCLs (22 PTL patients and 232 nodal DLBCLs patients from GSE10524，GSE10846，GSE61578) [file 12967_2024_5140_MOESM8_ESM.pdf]

Supplementary Figure 11

A

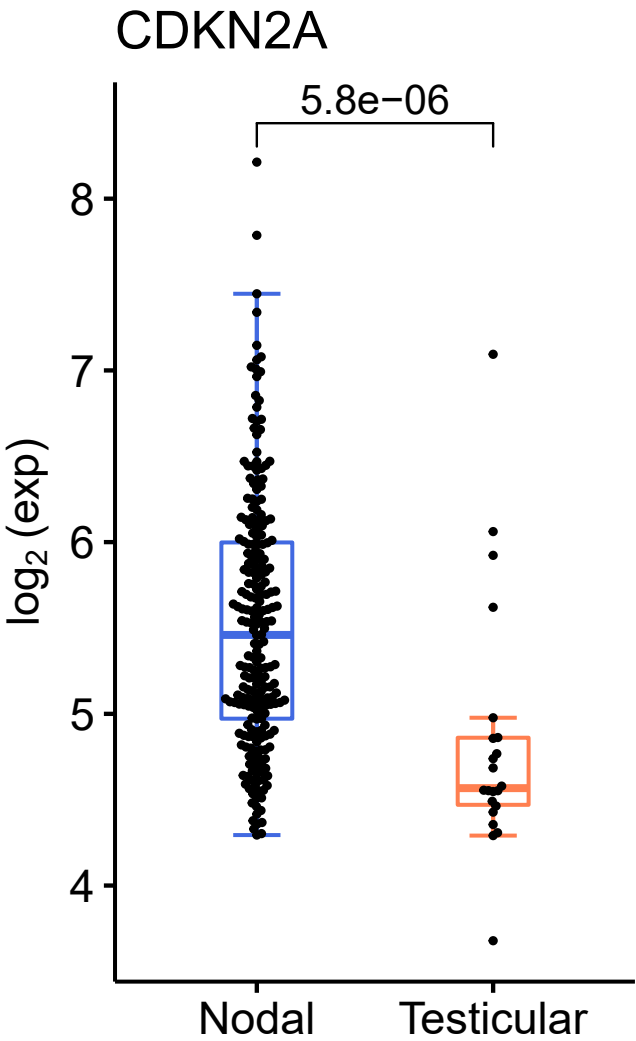

Supplement: Supplementary file 11 — Additional file 11: Figure S11. Different gene expression between PTL patients and nodal DLBCLs. (A) The expression of CDKN2A between PTL patients and nodal DLBCLs. [file 12967_2024_5140_MOESM11_ESM.pdf]
