# Supplementary material for: Genomic landscape and distinct molecular subtypes of primary testicular lymphoma
Source: J Transl Med. 2024 May 1;22:414. doi: 10.1186/s12967-024-05140-8 (PMC11064289; doi:10.1186/s12967-024-05140-8)

Supplementary Figure 9

A

Gene	Amp(Del)	P.value
P4HTM	0.08 (0.24)	7.6e−03
WDR6	0.08 (0.24)	7.6e−03
NDUFAF3	0.08 (0.24)	7.6e−03
DALRD3	0.08 (0.24)	7.6e−03
IMPDH2	0.08 (0.24)	7.6e−03
TREX1	0.08 (0.24)	3.0e−02
SHISA5	0.08 (0.24)	3.0e−02
PFKFB4	0.08 (0.24)	3.0e−02
UCN2	0.08 (0.24)	3.0e−02

B

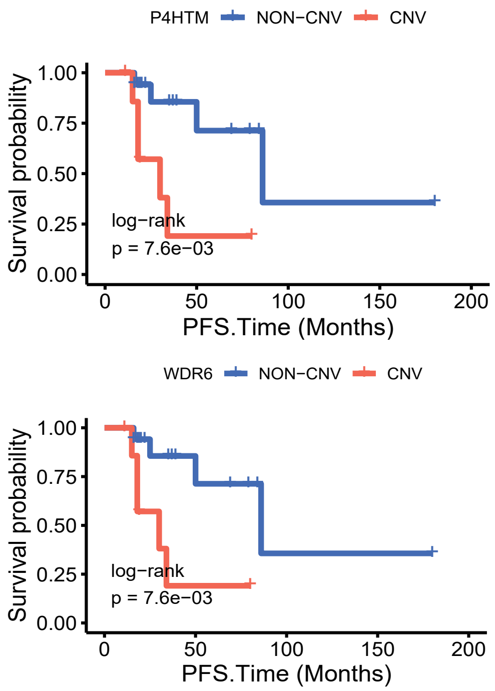

Supplement: Supplementary file 9 — Additional file 9: Figure S9. Effect of deletion-based genes on prognosis in PTL patients. A Forest plots of deleted genes. The Kaplan-Meier curves for PFS of the CNV in P4HTM (log-rank test, P=7.6e−03) and WDR6 (log-rank test, P=7.6e−03). [file 12967_2024_5140_MOESM9_ESM.pdf]
