# Supplementary material for: Genomic landscape and distinct molecular subtypes of primary testicular lymphoma
Source: J Transl Med. 2024 May 1;22:414. doi: 10.1186/s12967-024-05140-8 (PMC11064289; doi:10.1186/s12967-024-05140-8)

Supplementary Figure 10

A

Gene	Amp(Del)	P.value
TLE1	0.2 (0.08)	9.0e−04
ADAMTSL4−AS1	0.2 (0.04)	3.0e−03
EIF3B	0.2 (0.04)	4.6e−03
DNAAF5	0.28 (0.04)	6.4e−03
SUN1	0.28 (0.04)	6.4e−03
GET4	0.28 (0.04)	6.4e−03
CYP2W1	0.28 (0.04)	6.4e−03
GPR146	0.28 (0.04)	6.4e−03
GPER1	0.28 (0.04)	6.4e−03
UNCX	0.28 (0.04)	6.4e−03
PDGFA	0.28 (0.04)	6.4e−03
PRKAR1B	0.28 (0.04)	6.4e−03
ADAP1	0.28 (0.04)	6.4e−03
COX19	0.28 (0.04)	6.4e−03
C7orf50	0.28 (0.04)	6.4e−03
ZFAND2A	0.28 (0.04)	6.4e−03
MICALL2	0.24 (0.04)	6.4e−03
MAFK	0.28 (0.04)	8.4e−03
ELFN1	0.28 (0.04)	8.4e−03
NUDT1	0.28 (0.04)	8.4e−03
INTS1	0.28 (0.04)	8.4e−03
TMEM184A	0.28 (0.04)	8.4e−03
PSMG3	0.28 (0.04)	8.4e−03
MAD1L1	0.28 (0.04)	8.4e−03
MRM2	0.28 (0.04)	8.4e−03
SNX8	0.28 (0.04)	8.4e−03
MADCAM1	0.16 (0.08)	9.9e−03
MIER2	0.16 (0.08)	9.9e−03
THEG	0.16 (0.08)	9.9e−03
C2CD4C	0.16 (0.08)	9.9e−03
SHC2	0.16 (0.08)	9.9e−03
ODF3L2	0.16 (0.08)	9.9e−03
VPS45	0.2 (0.04)	1.3e−02
PLEKHO1	0.2 (0.04)	1.3e−02
SF3B4	0.2 (0.04)	1.3e−02
MTMR11	0.2 (0.04)	1.3e−02
OTUD7B	0.2 (0.04)	1.3e−02

B

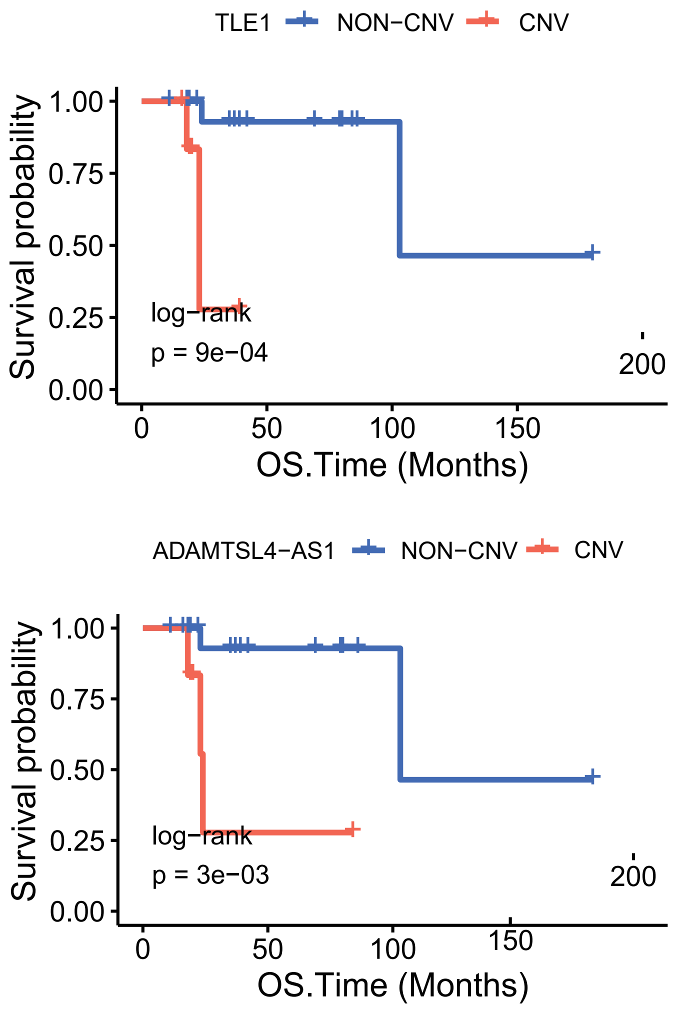

Supplement: Supplementary file 10 — Additional file 10: Figure S10. Effect of amplification-based genes on prognosis in patients with PTL. A Forest plots of amplified genes. B The Kaplan-Meier curves for OS of the CNV in TLE1 (log-rank test, P=9e−04) and ADAMTSL4-AS1 (log-rank test, P=3e−03) [file 12967_2024_5140_MOESM10_ESM.pdf]
